# Prostate Biopsy Once Again Is a Hot Topic in This Issue of the International Brazilian Journal of Urology

**DOI:** 10.1590/S1677-5538.IBJU.2026.03.01

**Published:** 2026-04-09

**Authors:** Luciano A. Favorito

**Affiliations:** 1 Universidade do Estado do Rio de Janeiro Unidade de Pesquisa Urogenital Rio de Janeiro RJ Brasil Unidade de Pesquisa Urogenital - Universidade do Estado do Rio de Janeiro - Uerj, Rio de Janeiro, RJ, Brasil; 2 Hospital Federal da Lagoa Serviço de Urologia Rio de Janeiro RJ Brasil Serviço de Urologia, Hospital Federal da Lagoa, Rio de Janeiro, RJ, Brasil

The May-June number of International Brazilian Journal of Urology presents original contributions with a lot of interesting papers in different fields: Prostate cancer, Prostate biopsy, Male hypogonadism, Uro-Radiology, Posterior Urethral Valves, Transplant, Robotic Surgery, Kidney cancer, Infertility, Prostate Cancer and Reconstructive Urology. The papers came from many different countries such as Brazil, Italy, China, Egypt, USA, Portugal and Turkey, and as usual the editor's comment highlights some of them. The editor in chief would like to highlight the works about Prostate biopsy.

Dr. Chua and collegues from USA, presented in page e20250512 (1) a nice study about the Pain Perception During Transperineal and Transrectal Prostate Biopsy (PBx) Under Local Anesthesia (LA) and concluded that PBx under LA alone are generally well tolerated; however, there is a subset of patients who experience more pain, including Black and Latino, younger patients, and those with more MRI suspicious lesions. Discussion of these pain risk factors is important for pa- tients when choosing to have a biopsy performed under LA versus sedation.

Dr. Pina and collegues from Portugal, presented in page e20250653 (2) a interesting study about systematic biopsy (SB) after targeted biopsy for the detection of clinically significant prostate cancer in MRI and concluded that SB offers limited diagnostic value when combined with a mpMRI-targeted approach and support the latter as a stand-alone procedure in men with suspicious lesions.

Prostate biopsy has been showing great prominence in diagnosis of prostate cancer recently (3–6). These 2 studies are very interesting and will greatly assist the urologist in diagnosing this disease.

The Editor-in-chief expects everyone to enjoy reading.
